# Anti-inflammatory effect of interleukin-6 highly enriched in secretome of two clinically relevant sources of mesenchymal stromal cells

**DOI:** 10.3389/fcell.2023.1244120

**Published:** 2023-09-07

**Authors:** Marianne Dedier, Brice Magne, Muriel Nivet, Sébastien Banzet, Marina Trouillas

**Affiliations:** ^1^ French Armed-forces Biomedical Research Institute (IRBA), Clamart, France; ^2^ UMR-MD-U1197, Inserm, Villejuif, France

**Keywords:** mesenchymal stromal cells, priming, extracellular vesicles, secretome, interleurkin-6, wound healing

## Abstract

Despite several advances in the field of regenerative medicine, clinical management of extensive skin wounds or burns remains a major therapeutic issue. During the past few years, Mesenchymal Stromal Cells (MSCs) have emerged as a novel therapeutic tool to promote tissue repair through their anti-inflammatory, pro-trophic and pro-remodeling effects. They exert their biological activity mainly via the secretion of soluble bioactive molecules such as cytokines, growth factors, proteins and microRNAs which can be encapsulated within extracellular vesicles (EV). The recent discovery of their high plasticity to external stimuli has fostered the development of new targeted therapies known as priming strategies, to enhance their potential. Our team recently showed that Interleukin-1β (IL-1β)-primed gingival MSCs promote wound healing and epidermal engraftment *in vitro*, and *in vivo* through their secreted products that contain extracellular vesicles. In the present work, we investigated whether two common sources of MSCs, gingiva and bone marrow, could respond similarly to IL-1β to favor pro-healing capabilities of their secretome. We showed that both primed-MSC sources, or their related secreted products, are able to reduce inflammation in LPS-challenged human monocytic THP-1 cell line. IL-1β priming enhanced MSC secretion of wound healing-related growth factors, cytokines and miRNAs in both sources. Among them, interleukin 6 was shown to be involved in the anti-inflammatory effect of MSC secreted products. Overall, these results underline the pro-healing properties of both MSC sources and their secretome upon IL-1β priming and their potential to improve the current medical treatment of severe wounds.

## 1 Introduction

Restoration of the integrity and function of wounded skin is finely orchestrated in inflammation, proliferation and remodeling phases involving specialized cell types, growth factors and cytokines (Gurtner et al., 2008). For example, macrophages play key roles in the inflammatory-proliferative phase transition, but these versatile cells also assist in all other stages of wound healing, to promote successful repair (Koh and DiPietro, 2011). Abnormal macrophage function and excessive inflammation, seen in chronic diseases or important acute traumas, may result in delayed healing or excessive scarring (Landen et al., 2016; Xu et al., 2020).

In recent years, many studies have highlighted the interesting repair properties of mesenchymal stromal cells (MSC) (Cerqueira et al., 2016; Nourian Dehkordi et al., 2019). Preclinical studies have shown that MSCs influence distinct phases of wound healing, including macrophage polarization, acceleration of reepithelialization and matrix remodeling (Jackson et al., 2012; Chen et al., 2016). Early clinical data have also reported their safety and efficacy for wound repair (Bey et al., 2010; Huang et al., 2020). Eventually, numerous preclinical studies have emphasized the growing interest of their secretory products, especially their extracellular vesicles (EV), for skin repair, which paved the way for future acellular therapies (Chen et al., 2008; Li et al., 2016; Kucharzewski et al., 2019).

Since their discovery in the bone marrow, MSCs have been isolated from numerous tissues such as perinatal tissues, dental pulp or adipose tissues. However, MSC phenotype, biological characteristics, and secretory activities differ according to their tissue sources (Macrin et al., 2017). Indeed, it has been shown that gingiva-derived MSCs (G-MSCs) possess better proliferation and migration capacity than adipose-derived MSCs (Boink et al., 2015), and that bone marrow-derived MSCs (BM-MSCs) display a higher immunomodulatory activity compared to adipose or Wharton’s jelly-derived MSCs (Petrenko et al., 2020). There is currently no source commonly accepted for wound healing therapy. G-MSCs and BM-MSCs are two sources with interesting wound healing-related properties. On the one hand, G-MSCs have been shown to enhance wound healing through anti-inflammatory and pro-remodeling properties in a burn irradiation model (Linard et al., 2015). On the other hand, BM-MSCs have been shown to increase re-epithelialization and thickness of the regenerated epidermis (Fu et al., 2006), and to accelerate wound closure through induction of macrophage polarization toward a pro-healing phenotype (Alapure et al., 2018). Moreover, both sources are known to produce EVs and growth factors with potent repair activities (Pires et al., 2016; Yamada et al., 2019; Magne et al., 2020; Nakao et al., 2021; Lorenzini et al., 2023).

MSCs are also known for their ability to sense their environment and adapt accordingly to molecular, cellular, and physical environments (Kusuma et al., 2017). This property has led to the use of priming cues as stimuli to enhance MSC activity (Madrigal et al., 2014). Upon injury, resident and remote cells are both recruited to the wound area and activated by inflammatory cytokines. Therefore, interferon gamma (IFN-γ), tumor necrosis factor alpha (TNF-α) and interleukin 1 beta (IL-1β) have been widely used to prime MSCs to a wounding environment (Noronha et al., 2019). In our previous work, we showed that IL-1β-primed G-MSCs accelerate wound healing through the modification of their secretory profile (Magne et al., 2020). Several studies indicate that pure EVs are not sufficient to recapitulate MSC properties (Mitchell et al., 2019; Papait et al., 2022; Wolf et al., 2022). Soluble molecules can non-covalently bind around EVs forming a corona which itself can participate in the biological effects of MSC-secreted products (Wolf et al., 2022). However, the impact of an inflammatory priming on the secretome of different MSC sources and their efficacy to treat skin wound healing has not been explored.

Here, we aimed to compare the impact of IL-1β priming on the anti-inflammatory properties of two different MSC sources and their secretomes. Our results demonstrated that both sources can decrease inflammation. We also found that IL-1β priming modify the secretome (proteins and miRNAs) of both cell types. In particular, interleukin 6 (IL-6) is significantly highly secreted upon priming and plays a substantial part in the reduction of inflammation induced by MSC secretions.

## 2 Materials and methods

### 2.1 Cell isolation, culture and characterization

This study was conducted in accordance with ethical principles stated in the declaration of Helsinki. All the human cells were isolated from surgical residues collected from healthy subjects (surgical stomatology for G-MSC, hip replacement surgery for BM-MSC). An informed, written consent was obtained from donors. According to French law, a declaration but no ethical committee approval was required for using these samples.

G- and BM-MSC were harvested as described previously (Doucet et al., 2005; Magne et al., 2020) and were cultivated in MEMα (Sartorius) culture medium supplemented with 5% human platelet lysate [French Armed-forces Blood Transfusion Center (Doucet et al., 2005)], 2 IU/mL heparin (Sanofi) and 100 IU/mL penicillin (Panpharma), 50 μg/mL gentamicin (Panpharma), and 1 μg/mL amphotericin B (Cheplapharm Arzneimittel GmbH) or 10 μg/mL Ciprofloxacin (Bayer) for G- or BM-MSC respectively, at 37°C in a humid atmosphere under 5% CO_2_. G- and BM-MSCs were characterized by flow cytometry and differentiation assays as recommended by ISCT guidelines (Doucet et al., 2005; Dominici et al., 2006; Magne et al., 2020).

The human monocytic THP-1 cell line (ATCC) was cultured in RPMI (Gibco) medium supplemented with 10% heat-inactivated fetal bovine serum (Cytiva), 50 µM β-mercapto-ethanol (Sigma) and penicillin-streptomycin (100 U/mL and 100 μg/mL respectively, Gibco).

### 2.2 MSC priming and conditioned medium preparation

Following a previously-described method (Magne et al., 2020), 7 individual cell populations of either G-MSCs or BM-MSCs were grown at passage 4 until 60% confluence and primed for 24 h with 1 ng/mL human recombinant IL-1β (Peprotech) (MSC_IL_) or no treatment (MSC_NV_). All seven populations of either G-MSCs or BM-MSCs were grown and primed separately, but were used together in functional assays after pooling them in equal cell quantities. For conditioned media (CM) preparation, all seven populations of G-MSCs or BM-MSCs were grown and primed separately, washed three times in phosphate-buffered saline (PBS, Thermo fisher) and incubated in human platelet lysate- and antibiotic-free medium for 48 h. Conditioned media from G-MSCs (G-CM) and BM-MSCs (BM-CM), either untreated (CM_NV_) or after priming (CM_IL_) were separately collected and concentrated 40 times using Amicon ultra centrifugal filter units with 3-kDa cutoff (Millipore). In functional assays, all individual CM_NV_ and CM_IL_ for both G-MSCs and BM-MSCs were pooled together in equal volumes. Pooled CM total protein amount was determined using the Bio-Rad Protein Assay kit. The contents of each individual CM were analyzed using enzyme-linked immunosorbant assays (ELISAs) according to manufacturer’s instructions (Biotechne) and real time quantitative polymerase chain reaction (RT-qPCR, Section 2.4).

### 2.3 Inflammation assay

Human monocytic THP-1 cells were seeded in 24-well plates at 170,000 cells/mL, exposed to 1 μg/mL Lipopolysaccharide (LPS; *Escherichia coli* O55:B5, L6529; Sigma), and cultured with either pooled MSC_NV_ or MSC_IL_ at a 1:10 MSC-to-THP-1 ratio, pooled CM_NV_ or CM_IL_ at 10 μg/mL of total proteins, or human recombinant IL-6 (Biotechne) at 695 pg/mL (corresponds to the concentration measured in BM-CM_IL_). In some experiments, the THP-1 cells were also treated either with an IL-6 receptor blocking antibody (Tocilizumab, Selleckchem) or its IgG1 isotypic control (Biotechne) at 100 μg/mL. THP-1 supernatants were collected after 24 h and assayed for TNF-α and interleukin 1 receptor antagonist (IL-1RA) levels by ELISAs (Biotechne).

### 2.4 RT-qPCR

A volume of 100 µL of individual CM was mixed first with 300 µL Trizol LS Reagent (Sigma) and then with 80 µL chloroform (Sigma) according to the manufacturer’s protocol (Sigma). Next, aqueous phases were mixed with 200 µL isopropanol (Sigma) and 1 µL of glycoblue (Ambion AM9515). After 10 min of incubation, the preparations were centrifuged at 12,000 g for 10 min. The pellets were washed with 75% ethanol (Sigma), air-dried, and resuspended in 20 µL of RNAse free water (Qiagen). The samples were incubated at 55°C during 15 min and frozen for future experiments.

Total RNA (10 ng per sample) was transcribed into cDNA using miRCURY LNA RT Kit for miRNA (Qiagen). RT-qPCRs were carried out using miRCURY LNA miRNA PCR Assays according to manufacturer’s protocol (Qiagen) with LightCycler 480 II (Roche Diagnostics), and analyzed with the LightCycler software (Roche Diagnostics). The expressions of the target miRNAs were normalized with the geometric mean of 4 reference miRNAs (miR-191-5p, miR-23a-3p, miR-100-5p, miR-199a-3p) selected using GeNorm software V3.5 (Vandesompele et al., 2002). Final quantification is expressed as arbitrary units (AU) and consists of the geometrical mean of the relative quantification (2^−ΔΔCT^) performed with each reference miRNA.

### 2.5 Statistics

All statistical analyses were conducted on R software (v. 4.0.2). Wilcoxon or Mann-Whitney tests were performed to compared two conditions. For the variables having more than 2 modalities, we performed Kruskal–Wallis test and Wilcoxon *post hoc* test with *p* values adjusted by the FDR method. Significance level was set to *p* < 0.05. All charts were plotted as mean ± standard error of the mean on GraphPad Prism 6 software.

## 3 Results

### 3.1 Both MSC sources have comparable anti-inflammatory effects *in vitro*

**FIGURE 1:**
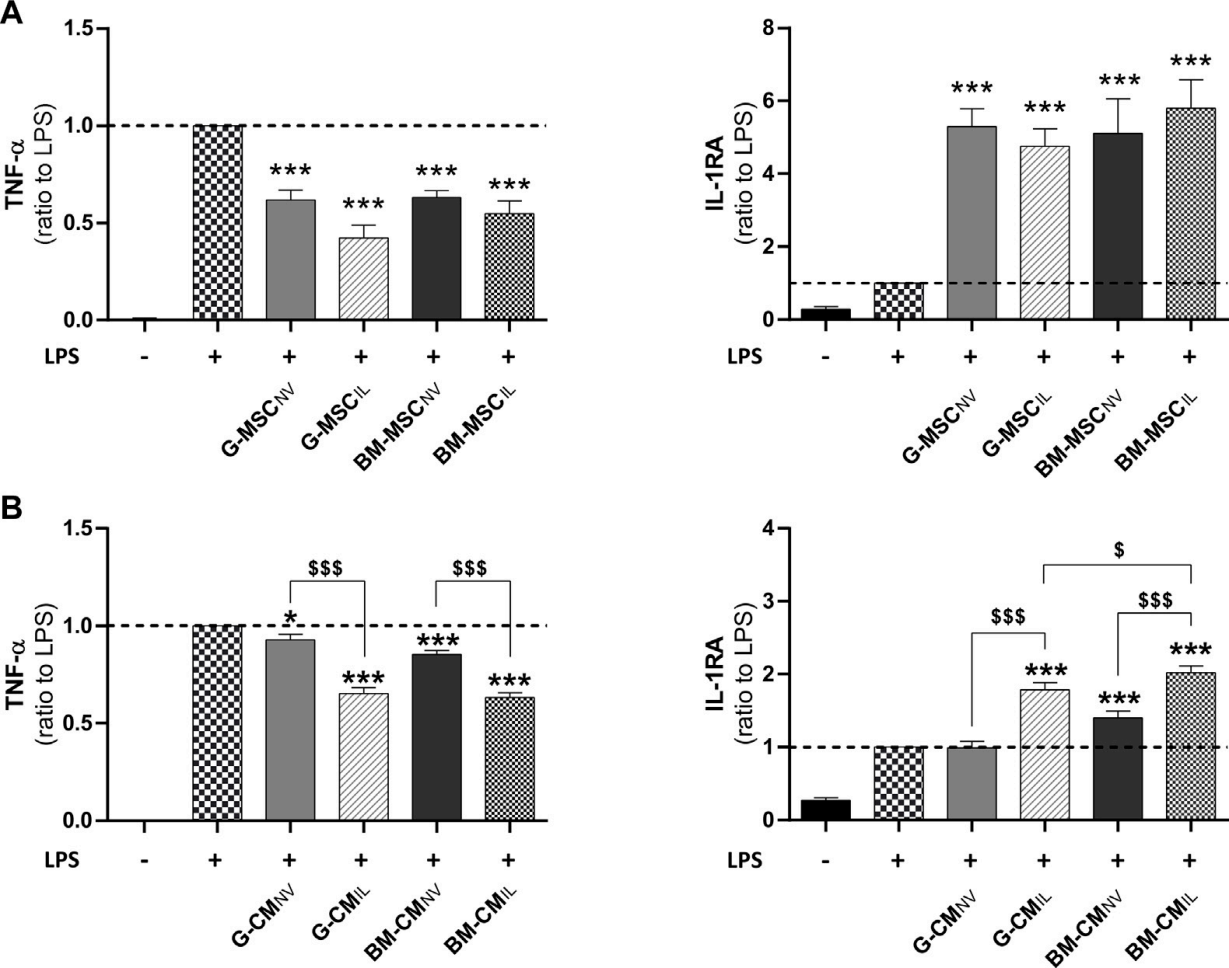
Bone marrow and gingiva-derived MSCs display comparable anti-inflammatory effects *in vitro*. **(A)** Dosage of TNF-α and IL-1RA in the supernatants of LPS-challenged THP-1 cells co-cultured for 24 h with BM or G-MSC_NV_ or MSC_IL_ (ratio to LPS, *n* = 5–10). **(B)** Dosage of TNF-α and IL-1RA in the supernatants of LPS-challenged THP-1 cells cultured with BM or G-CM_NV_ or CM_IL_ at 10 μg/mL of total proteins for 24 h (ratio to LPS, *n* = 9–19). Values are expressed as mean ± SEM. *p < 0.05, ***p < 0.001 (comparison with LPS alone condition); $ *p* < 0.05, $$$ *p* < 0.001 (comparison between indicated conditions); SEM, standard error of the mean; MSC_NV_, naive mesenchymal stromal cells; MSC_IL_, IL-1β-primed MSC; BM, bone marrow, G, gingiva; CM, conditioned medium; IL-1RA, IL-1 receptor antagonist; LPS, lipopolysaccharide; TNF-α, tumor necrosis factor-α.

We investigated the immunomodulation response of BM-MSCs and G-MSCs after IL-1β-priming, using an *in vitro* inflammation assay. First, MSC_NV_ and MSC_IL_ from bone marrow and gingiva both reduced the inflammatory response of LPS-challenged human monocytic THP-1 cell line as demonstrated by a strong decrease in TNF-α and increase in IL-1RA levels in supernatants (*p* < 0.001, Figure 1A). MSC_IL_ tended to be superior to MSC_NV_ in decreasing TNF-α levels in THP-1 supernatants, for the gingiva origin (*p* = 0.105, Figure 1A) compared to bone marrow (*p* = 0.357, Figure 1A). We next investigated whether similar effects would be observed using concentrated CM of MSCs from both sources. CM concentration was performed to increase the concentration of the soluble factors and EVs in CM (Magne et al., 2020). We showed that CM_IL_ were more efficient to decrease TNF-α and increase IL-1RA compared to CM_NV_ in both sources in LPS-challenged THP-1 supernatants (*p* < 0.001, Figure 1B). We also noted that BM-CM_IL_ induced a higher increase of IL-1RA compared to G-CM_IL_ (*p* < 0.05, Figure 1B). Thus, these results indicate a similar anti-inflammatory response for both MSC sources and their respective secretome upon IL-1β priming.

### 3.2 IL-1β priming impacts MSC secretome of both sources

**FIGURE 2:**
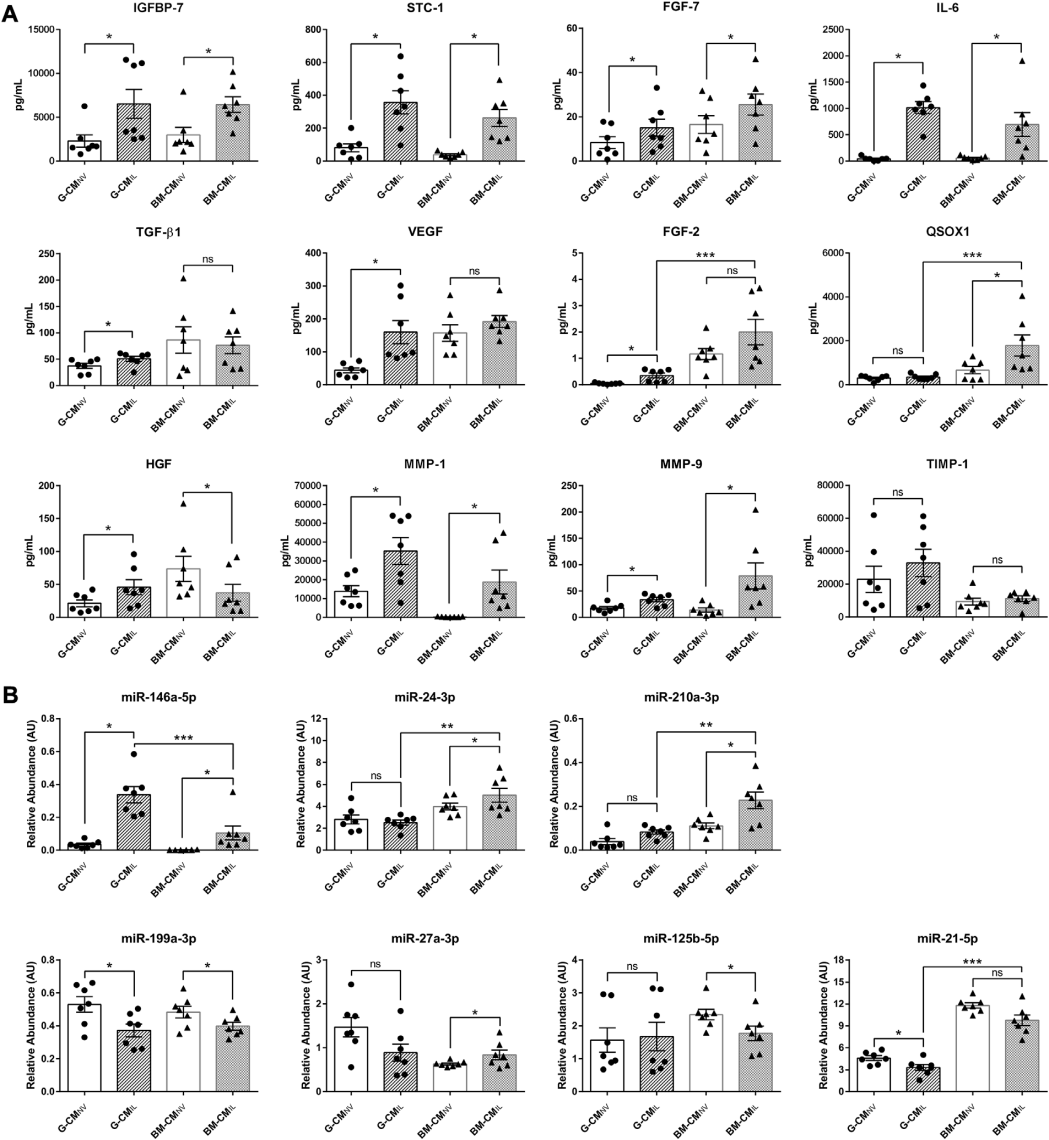
IL-1β priming remodel the secretome of bone marrow and gingiva-derived MSCs. **(A)** ELISA dosages of selected wound healing-related proteins in CM_NV_ or CM_IL_ of both sources G or BM used at 10 μg/mL of total proteins (*n* = 7 MSC donors). **(B)** Relative abundance of selected miRNA in CM_NV_ or CM_IL_ of both sources of MSCs normalized with four internal reference miRNA (*n* = 7 MSC donors). Values are expressed as mean ± SEM. *p < 0.05; **p < 0.01; ***p < 0.001; ns, non-significant; CM, conditioned medium; G, gingival; BM, bone marrow; NV, naive; IL, primed with IL-1β; AU, arbitrary units; IGFBP-7, insulin growth factor protein 7; STC-1, stanniocalcin-1; FGF-7, fibroblast growth factor-7; IL-6, interleukin-6; TGF-β1; transforming growth factor β1; VEGF, vascular endothelial growth factor; FGF-2, fibroblast growth factor 2; QSOX1, quiescin sulfhydryl oxidase 1; HGF, hepatocyte growth factor; TIMP-1, tissue inhibitor of metalloproteinases 1; MMP-1, matrix metalloproteinase 1; MMP-9, matrix metalloproteinase 9.

As MSC efficiency relies on release of secretory products (Nourian Dehkordi et al., 2019), we next sought to better understand their mechanisms of action. Therefore, we quantified by ELISA a selection of wound healing-related proteins known to be key paracrine actors of the MSC effects in our CM, to determine if both sources respond similarly to IL-1β. We found that IGFBP-7, STC-1, FGF-7, and IL-6 were significantly upregulated in CM_IL_ compared to CM_NV_ in both sources (*p* < 0.05, Figure 2A). The IL-1β priming significantly increased TGF-β1, VEGF and FGF-2 secretion (*p* < 0.05, Figure 2A) only in the G-CM whereas QSOX1 secretion was significantly increased only in the BM-CM (*p* < 0.05, Figure 2A). Priming induced different HGF secretion responses in both sources, with an increase in HGF expression in the G-CM_IL_ and a decrease in HGF levels in the BM-CM_IL_ (*p* < 0.05, Figure 2A). Molecules involved in extracellular matrix remodeling were also affected by the priming in both sources, with a significant increase in matrix metallopeptidase 1 (MMP-1) and MMP-9 secretion (*p* < 0.05, Figure 2A). However, IL-1β priming had no impact on tissue inhibitor metallopeptidase 1 (TIMP-1) secretion (Figure 2A). When comparing CM_IL_ from both sources, our results indicated that BM-CM contained a significantly higher concentration of QSOX1 and FGF-2 compared to G-CM (*p* < 0.05, Figure 2A). We also studied the secretion of a selection of miRNAs specifically related to wound healing functions (Fang et al., 2016; Hu et al., 2018). Our results indicated a differential regulation of macrophage polarization-related miRNAs (Li H. et al., 2018) between both MSC sources after IL-1β priming. Concerning M2-polarizing miRNAs, we showed an upregulation of miR-146a-5p in CM_IL_ compared to CM_NV_, in both sources (*p* < 0.05, Figure 2B). The secretion of miR-24-3p and miR-210a-3p were only upregulated in CM_IL_ for BM source (*p* < 0.05, Figure 2B). Concerning M1-related miRNA, we found a decrease of miR-199a-3p in CM_IL_ compared to CM_NV_ in both sources (*p* < 0.05, Figure 2B). MiR-21-5p was decreased only in G-CM_IL_ whereas miR-125b-5p secretion was decreased only in BM-CM_IL_ (*p* < 0.05, Figure 2B). When comparing CM_IL_ from both sources, our results indicated that G-CM contained a significantly higher concentration of miR-146a-5p (*p* < 0.001, Figure 2B) whereas BM-CM contained a significantly higher concentration of miR-24-3p (*p* < 0.01), miR-210-3p (*p* < 0.01), and miR-21-5p (*p* < 0.001, Figure 2B). Thus, these results indicate slight differences between the two secretory profiles among both sources with higher secretion of wound healing-related molecules in BM-MSC_IL_.

### 3.3 IL-6 plays key roles in the anti-inflammatory effect of CM from both MSC sources

**FIGURE 3:**
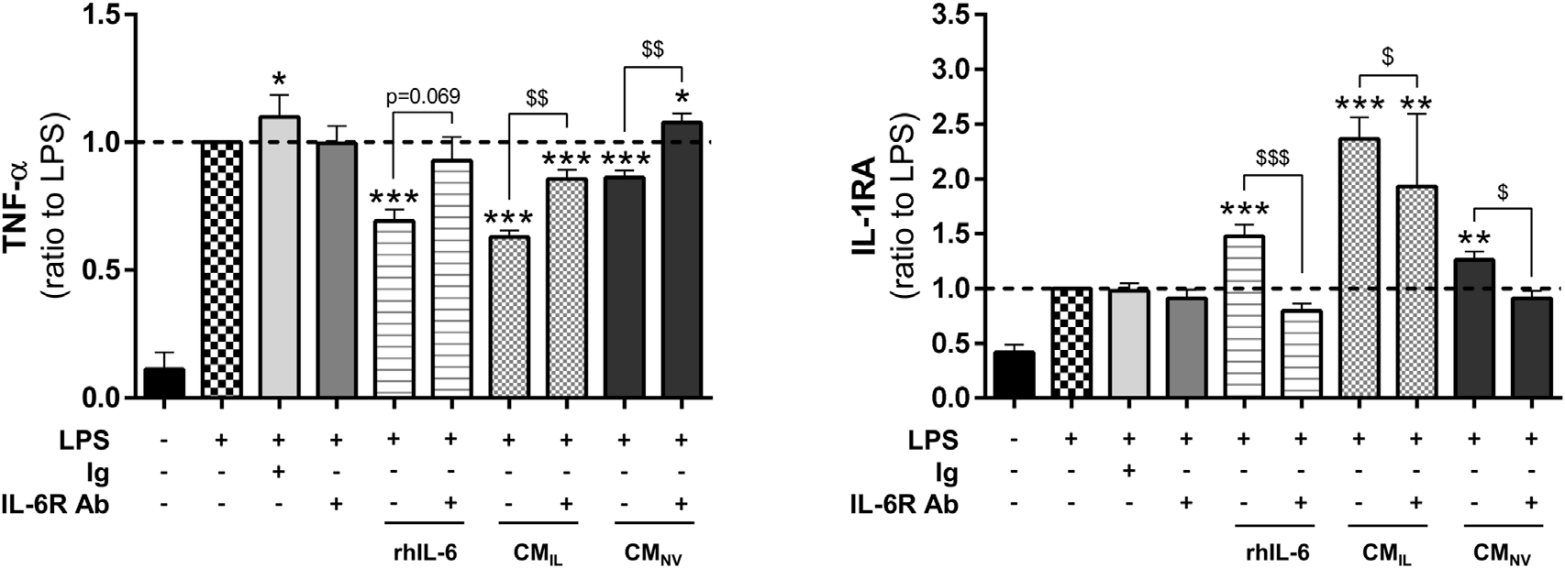
IL-6 plays a key role in the anti-inflammatory effect of CM from bone marrow and gingiva-derived MSCs. Dosage of TNF-α and IL-1RA in the supernatants of LPS-challenged THP-1 cells with rhIL-6 at the concentration found in BM-CM_IL_, BM-CM_IL_ or BM-CM_NV_ with or without IL-6 antibody or control antibody (ratio to LPS, *n* = 5–14) for 24 h. Values are expressed as mean ± SEM. *p < 0.05; **p < 0,01; ***p < 0.001 (comparison with LPS alone condition); $ *p* < 0.05; $$ *p* < 0,01; $$$ *p* < 0.001 (comparison between indicated conditions); SEM, standard error of the mean; IL-1RA, IL-1 receptor antagonist; LPS, lipopolysaccharide; TNF-α, tumor necrosis factor-α; CM_NV_, conditioned medium from naive MSC; CM_IL_ conditioned medium from IL-1β-primed MSC; IL-6R Ab, IL-6 receptor antibody; Ig, control antibody; rhIL-6, recombinant IL-6.

In our secretome analysis (Figure 2), IL-6 appears to be one of the factors whose concentration is most increased during IL-1β priming. According to the literature, IL-6 plays important roles in wound healing (McFarland-Mancini et al., 2010). We thus investigated whether IL-6 could be responsible for the anti-inflammatory effect elicited by the CM_IL_ in our inflammation assay. Similarly to BM-CM_IL_, recombinant human IL-6 (rhIL-6), used alone at the concentration measured in BM-CM_IL_, significantly decreased TNF-α levels in LPS-challenged THP-1 cell cultures compared to LPS (*p* < 0.001, Figure 3) and increased IL-1RA (*p* < 0.001, Figure 3). Similar results were obtained when using rhIL-6 alone at the concentration measured in G-CM_IL_ (data not shown). The use of IL-6 receptor blocking antibody partly counteracted the effect of rhIL-6, BM-CM_IL_ and BM-CM_NV_ on TNF-α secretion (*p* = 0.069 for rhIL-6, *p* < 0.01 for BM-CM_IL_ and BM-CM_NV_, Figure 3) and also on IL-1 RA secretion (*p* < 0.05 for rhIL-6, *p* < 0.001 for BM-CM_IL_ and BM-CM_NV_, Figure 3). Taken together, these results suggest that BM-CM_IL_ promote reduction of inflammation in part through IL-6 pathway.

## 4 Discussion

MSCs hold great potential in cell therapy for skin wound healing due to their immunomodulatory properties, their pleiotropic activities to support other cell types and their capacity to respond to their environment. In this study, we tested whether MSCs from different tissue sources (including bone marrow and gingiva) can display similar anti-inflammatory potency and respond comparably to IL-1β priming. Our results indicate that both sources are comparable after IL-1β priming in our *in vitro* model of inflammation (Figures 1A, B). These findings correlate with previous *in vitro* studies showing no difference between both sources for their immunomodulatory ability in a T lymphocyte proliferation test (Li J. et al., 2018), or with *in vivo* study indicating similar effects of BM and G-MSCs to reduce necrosis in the stasis zone around the burn in a rat model (Abbas et al., 2019).

We also showed that IL-1β priming improves the anti-inflammatory effect of MSC secretome from both sources (Figure 1B and data not shown). This beneficial effect correlates with other studies evidencing that IL-1β-primed MSC-derived secretory products ameliorate sepsis through macrophage M2 polarization (Song et al., 2017; Yao et al., 2021) and favor wound healing of full-thickness excisional skin wounds (Park et al., 2018). Our study, along with others (Maffioli et al., 2017; Redondo-Castro et al., 2018), evidence that inflammatory priming leads to major secretome changes in terms of growth factors, interleukins, inflammatory mediators and extracellular matrix remodeling components. Moreover, studies comparing different inflammatory primings (Burja et al., 2020; Wedzinska et al., 2021), indicated that IL-1β most profoundly increases the expression of cytokines and chemokines with anti-inflammatory properties. Our results indicated that basal level in CM_NV_ of some factors (VEGF, HGF, MMP-1) were significantly different between both sources but became similar in CM_IL_. While some factors were differentially secreted upon IL-1β priming between the two sources, anti-inflammatory factors such as IGFBP-7 or IL-6 were strongly secreted in both sources (Figure 2A). As shown in several studies (Peltzer et al., 2020; Giunti et al., 2021), inflammatory priming impacts also the composition of EV cargoes such as miRNAs. Several mi-RNAs as miR-199a-3p, miR-125b-5p, miR-21-5p found in our study are also reported in miRNAs landscape of MSC-Extracellular Vesicles described by Ferguson (Ferguson et al., 2018), comforting that miRNA found in our secretome may be carried out by EVs. Several studies reported that miR-146a-5p, which is also upregulated after IL-1β priming, is capable to decrease inflammation and fibrosis formation (Song et al., 2017; Liang et al., 2019; Wu et al., 2019). Our study showed variations in the factors and miRNAs evaluated between both sources. For example, some factors with favorable ant-inflammatory activity reported in literature such as miR-146a-5p or FGF-2, QSOX-1 and miR-24-3p were found in higher amount in G-CM_IL_ or BM-CM_IL_ respectively. However, CM_IL_ from both sources displayed anti-inflammatory activity (Figure 1B) suggesting a balance provided by the amounts of the different factors. As other investigators, we showed that IL-1β induces the secretion of IL-6 in both MSC tissue sources (Burja et al., 2020). In our study, we demonstrated with a blocking antibody that IL-6 plays a key role in the anti-inflammatory properties of the CM (Figure 3). These results correlate with different studies shedding light on the potential role of IL-6 in wound healing. Heo et al. (2011) showed that a conditioned medium from TNF-α-primed MSCs stimulates cutaneous wound healing through IL-6- and IL-8-dependent mechanisms. Another team provided evidence that BM-MSC inflammatory preconditioning highly favors their potential to promote IL-6-dependent M2b polarization (Philipp et al., 2018). A cyto-protective effect of MSC-EV on liver failure has also been evidenced involving the IL-6/STAT3 pathway (Tan et al., 2014). Thus, the reported effects of CM_IL_ in our study could be attributable to the presence of proteins and EVs carrying growth factors, cytokines, and miRNAs.

In conclusion, in this article, both sources of primed-MSCs exhibit similar pro-healing properties. The priming strategy represents an interesting option to optimize the properties of MSC secretory products and possibly extracellular vesicles that could be used as a promising skin wound healing therapy.

## Data Availability

The original contributions presented in the study are included in the article/supplementary material, further inquiries can be directed to the corresponding author.

## References

[B1] Abbas O. L. Ozatik O. Gonen Z. B. Ogut S. Ozatik F. Y. Salkin H. (2019). Comparative analysis of mesenchymal stem cells from bone marrow, adipose tissue, and dental pulp as sources of cell therapy for zone of stasis burns. J. Invest. Surg. 32 (6), 477–490. 10.1080/08941939.2018.1433254 29442525

[B2] Alapure B. V. Lu Y. He M. Chu C. C. Peng H. Muhale F. (2018). Accelerate healing of severe burn wounds by mouse bone marrow mesenchymal stem cell-seeded biodegradable hydrogel scaffold synthesized from arginine-based poly(ester amide) and chitosan. Stem Cells Dev. 27 (23), 1605–1620. 10.1089/scd.2018.0106 30215325 PMC6276600

[B3] Bey E. Prat M. Duhamel P. Benderitter M. Brachet M. Trompier F. (2010). Emerging therapy for improving wound repair of severe radiation burns using local bone marrow-derived stem cell administrations. Wound Repair Regen. 18 (1), 50–58. 10.1111/j.1524-475X.2009.00562.x 20082681

[B4] Boink M. A. van den Broek L. J. Roffel S. Nazmi K. Bolscher J. G. M. Gefen A. (2015). Different wound healing properties of dermis, adipose, and gingiva mesenchymal stromal cells. Wound Repair Regen. 24, 100–109. 10.1111/wrr.12380 26542883

[B5] Burja B. Barlic A. Erman A. Mrak-Poljsak K. Tomsic M. Sodin-Semrl S. (2020). Human mesenchymal stromal cells from different tissues exhibit unique responses to different inflammatory stimuli. Curr. Res. Transl. Med. 68 (4), 217–224. 10.1016/j.retram.2020.05.006 32843323

[B6] Cerqueira M. T. Pirraco R. P. Marques A. P. (2016). Stem cells in skin wound healing: are we there yet? Adv. Wound Care (New Rochelle) 5 (4), 164–175. 10.1089/wound.2014.0607 27076994 PMC4817598

[B7] Chen D. Hao H. Fu X. Han W. (2016). Insight into reepithelialization: how do mesenchymal stem cells perform? Stem Cells Int. 2016, 6120173. 10.1155/2016/6120173 26770209 PMC4684897

[B8] Chen L. Tredget E. E. Wu P. Y. Wu Y. (2008). Paracrine factors of mesenchymal stem cells recruit macrophages and endothelial lineage cells and enhance wound healing. PLoS One 3 (4), e1886. 10.1371/journal.pone.0001886 18382669 PMC2270908

[B9] Dominici M. Le Blanc K. Mueller I. Slaper-Cortenbach I. Marini F. Krause D. (2006). Minimal criteria for defining multipotent mesenchymal stromal cells. The International Society for Cellular Therapy position statement. Cytotherapy 8 (4), 315–317. 10.1080/14653240600855905 16923606

[B10] Doucet C. Ernou I. Zhang Y. Llense J. R. Begot L. Holy X. (2005). Platelet lysates promote mesenchymal stem cell expansion: A safety substitute for animal serum in cell-based therapy applications. J. Cell Physiol. 205 (2), 228–236. 10.1002/jcp.20391 15887229

[B11] Fang S. Xu C. Zhang Y. Xue C. Yang C. Bi H. (2016). Umbilical cord-derived mesenchymal stem cell-derived exosomal MicroRNAs suppress myofibroblast differentiation by inhibiting the transforming growth factor-β/SMAD2 pathway during wound healing. Stem Cells Transl. Med. 5 (10), 1425–1439. 10.5966/sctm.2015-0367 27388239 PMC5031180

[B12] Ferguson S. W. Wang J. Lee C. J. Liu M. Neelamegham S. Canty J. M. (2018). The microRNA regulatory landscape of MSC-derived exosomes: A systems view. Sci. Rep. 8 (1), 1419. 10.1038/s41598-018-19581-x 29362496 PMC5780426

[B13] Fu X. Fang L. Li X. Cheng B. Sheng Z. (2006). Enhanced wound-healing quality with bone marrow mesenchymal stem cells autografting after skin injury. Wound Repair Regen. 14 (3), 325–335. 10.1111/j.1743-6109.2006.00128.x 16808812

[B14] Giunti D. Marini C. Parodi B. Usai C. Milanese M. Bonanno G. (2021). Role of miRNAs shuttled by mesenchymal stem cell-derived small extracellular vesicles in modulating neuroinflammation. Sci. Rep. 11 (1), 1740. 10.1038/s41598-021-81039-4 33462263 PMC7814007

[B15] Gurtner G. C. Werner S. Barrandon Y. Longaker M. T. (2008). Wound repair and regeneration. Nature 453 (7193), 314–321. 10.1038/nature07039 18480812

[B16] Heo S. C. Jeon E. S. Lee I. H. Kim H. S. Kim M. B. Kim J. H. (2011). Tumor necrosis factor-alpha-activated human adipose tissue-derived mesenchymal stem cells accelerate cutaneous wound healing through paracrine mechanisms. J. Invest. Dermatol 131 (7), 1559–1567. 10.1038/jid.2011.64 21451545

[B17] Hu Y. Rao S. S. Wang Z. X. Cao J. Tan Y. J. Luo J. (2018). Exosomes from human umbilical cord blood accelerate cutaneous wound healing through miR-21-3p-mediated promotion of angiogenesis and fibroblast function. Theranostics 8 (1), 169–184. 10.7150/thno.21234 29290800 PMC5743467

[B18] Huang Y. Z. Gou M. Da L. C. Zhang W. Q. Xie H. Q. (2020). Mesenchymal stem cells for chronic wound healing: current status of preclinical and clinical studies. Tissue Eng. Part B Rev. 26 (6), 555–570. 10.1089/ten.TEB.2019.0351 32242479

[B19] Jackson W. M. Nesti L. J. Tuan R. S. (2012). Concise review: clinical translation of wound healing therapies based on mesenchymal stem cells. Stem Cells Transl. Med. 1 (1), 44–50. 10.5966/sctm.2011-0024 23197639 PMC3727688

[B20] Koh T. J. DiPietro L. A. (2011). Inflammation and wound healing: the role of the macrophage. Expert Rev. Mol. Med. 13, e23. 10.1017/S1462399411001943 21740602 PMC3596046

[B21] Kucharzewski M. Rojczyk E. Wilemska-Kucharzewska K. Wilk R. Hudecki J. Los M. J. (2019). Novel trends in application of stem cells in skin wound healing. Eur. J. Pharmacol. 843, 307–315. 10.1016/j.ejphar.2018.12.012 30537490

[B22] Kusuma G. D. Carthew J. Lim R. Frith J. E. (2017). Effect of the microenvironment on mesenchymal stem cell paracrine signaling: opportunities to engineer the therapeutic effect. Stem Cells Dev. 26 (9), 617–631. 10.1089/scd.2016.0349 28186467

[B23] Landen N. X. Li D. Stahle M. (2016). Transition from inflammation to proliferation: A critical step during wound healing. Cell Mol. Life Sci. 73 (20), 3861–3885. 10.1007/s00018-016-2268-0 27180275 PMC5021733

[B24] Li H. Jiang T. Li M. Q. Zheng X. L. Zhao G. J. (2018a). Transcriptional regulation of macrophages polarization by MicroRNAs. Front. Immunol. 9, 1175. 10.3389/fimmu.2018.01175 29892301 PMC5985397

[B25] Li J. Xu S. Q. Zhao Y. M. Yu S. Ge L. H. Xu B. H. (2018b). Comparison of the biological characteristics of human mesenchymal stem cells derived from exfoliated deciduous teeth, bone marrow, gingival tissue, and umbilical cord. Mol. Med. Rep. 18 (6), 4969–4977. 10.3892/mmr.2018.9501 30272340 PMC6236220

[B26] Li X. Liu L. Yang J. Yu Y. Chai J. Wang L. (2016). Exosome derived from human umbilical cord mesenchymal stem cell mediates MiR-181c attenuating burn-induced excessive inflammation. EBioMedicine 8, 72–82. 10.1016/j.ebiom.2016.04.030 27428420 PMC4919539

[B27] Liang Y. C. Wu Y. P. Li X. D. Chen S. H. Ye X. J. Xue X. Y. (2019). TNF-alpha-induced exosomal miR-146a mediates mesenchymal stem cell-dependent suppression of urethral stricture. J. Cell Physiol. 234 (12), 23243–23255. 10.1002/jcp.28891 31144307

[B28] Linard C. Tissedre F. Busson E. Holler V. Leclerc T. Strup-Perrot C. (2015). Therapeutic potential of gingival fibroblasts for cutaneous radiation syndrome: comparison to bone marrow-mesenchymal stem cell grafts. Stem Cells Dev. 24 (10), 1182–1193. 10.1089/scd.2014.0486 25584741 PMC4425223

[B29] Lorenzini B. Peltzer J. Goulinet S. Rival B. Lataillade J. J. Uzan G. (2023). Producing vesicle-free cell culture additive for human cells extracellular vesicles manufacturing. J. Control Release 355, 501–514. 10.1016/j.jconrel.2023.01.073 36764527

[B30] Macrin D. Joseph J. P. Pillai A. A. Devi A. (2017). Eminent sources of adult mesenchymal stem cells and their therapeutic imminence. Stem Cell Rev. 13 (6), 741–756. 10.1007/s12015-017-9759-8 28812219

[B31] Madrigal M. Rao K. S. Riordan N. H. (2014). A review of therapeutic effects of mesenchymal stem cell secretions and induction of secretory modification by different culture methods. J. Transl. Med. 12, 260. 10.1186/s12967-014-0260-8 25304688 PMC4197270

[B32] Maffioli E. Nonnis S. Angioni R. Santagata F. Cali B. Zanotti L. (2017). Proteomic analysis of the secretome of human bone marrow-derived mesenchymal stem cells primed by pro-inflammatory cytokines. J. Proteomics 166, 115–126. 10.1016/j.jprot.2017.07.012 28739509

[B33] Magne B. Dedier M. Nivet M. Coulomb B. Banzet S. Lataillade J. J. (2020). IL-1β-Primed mesenchymal stromal cells improve epidermal substitute engraftment and wound healing via matrix metalloproteinases and transforming growth factor-β1. J. Invest. Dermatol 140 (3), 688–698. 10.1016/j.jid.2019.07.721 31513805

[B34] McFarland-Mancini M. M. Funk H. M. Paluch A. M. Zhou M. Giridhar P. V. Mercer C. A. (2010). Differences in wound healing in mice with deficiency of IL-6 versus IL-6 receptor. J. Immunol. 184 (12), 7219–7228. 10.4049/jimmunol.0901929 20483735

[B35] Mitchell R. Mellows B. Sheard J. Antonioli M. Kretz O. Chambers D. (2019). Secretome of adipose-derived mesenchymal stem cells promotes skeletal muscle regeneration through synergistic action of extracellular vesicle cargo and soluble proteins. Stem Cell Res. Ther. 10 (1), 116. 10.1186/s13287-019-1213-1 30953537 PMC6451311

[B36] Nakao Y. Fukuda T. Zhang Q. Sanui T. Shinjo T. Kou X. (2021). Exosomes from TNF-α-treated human gingiva-derived MSCs enhance M2 macrophage polarization and inhibit periodontal bone loss. Acta Biomater. 122, 306–324. 10.1016/j.actbio.2020.12.046 33359765 PMC7897289

[B37] Noronha N. C. Mizukami A. Caliari-Oliveira C. Cominal J. G. Rocha J. L. M. Covas D. T. (2019). Priming approaches to improve the efficacy of mesenchymal stromal cell-based therapies. Stem Cell Res. Ther. 10 (1), 131. 10.1186/s13287-019-1224-y 31046833 PMC6498654

[B38] Nourian Dehkordi A. Mirahmadi Babaheydari F. Chehelgerdi M. Raeisi Dehkordi S. (2019). Skin tissue engineering: wound healing based on stem-cell-based therapeutic strategies. Stem Cell Res. Ther. 10 (1), 111. 10.1186/s13287-019-1212-2 30922387 PMC6440165

[B39] Papait A. Ragni E. Cargnoni A. Vertua E. Romele P. Masserdotti A. (2022). Comparison of EV-free fraction, EVs, and total secretome of amniotic mesenchymal stromal cells for their immunomodulatory potential: A translational perspective. Front. Immunol. 13, 960909. 10.3389/fimmu.2022.960909 36052081 PMC9424831

[B40] Park S. R. Kim J. W. Jun H. S. Roh J. Y. Lee H. Y. Hong I. S. (2018). Stem cell secretome and its effect on cellular mechanisms relevant to wound healing. Mol. Ther. 26 (2), 606–617. 10.1016/j.ymthe.2017.09.023 29066165 PMC5835016

[B41] Peltzer J. Lund K. Goriot M. E. Grosbot M. Lataillade J. J. Mauduit P. (2020). Interferon-gamma and hypoxia priming have limited effect on the miRNA landscape of human mesenchymal stromal cells-derived extracellular vesicles. Front. Cell Dev. Biol. 8, 581436. 10.3389/fcell.2020.581436 33384991 PMC7769832

[B42] Petrenko Y. Vackova I. Kekulova K. Chudickova M. Koci Z. Turnovcova K. (2020). A comparative analysis of multipotent mesenchymal stromal cells derived from different sources, with a focus on neuroregenerative potential. Sci. Rep. 10 (1), 4290. 10.1038/s41598-020-61167-z 32152403 PMC7062771

[B43] Philipp D. Suhr L. Wahlers T. Choi Y. H. Paunel-Gorgulu A. (2018). Preconditioning of bone marrow-derived mesenchymal stem cells highly strengthens their potential to promote IL-6-dependent M2b polarization. Stem Cell Res. Ther. 9 (1), 286. 10.1186/s13287-018-1039-2 30359316 PMC6202843

[B44] Pires A. O. Mendes-Pinheiro B. Teixeira F. G. Anjo S. I. Ribeiro-Samy S. Gomes E. D. (2016). Unveiling the differences of secretome of human bone marrow mesenchymal stem cells, adipose tissue-derived stem cells, and human umbilical cord perivascular cells: A proteomic analysis. Stem Cells Dev. 25 (14), 1073–1083. 10.1089/scd.2016.0048 27226274

[B45] Redondo-Castro E. Cunningham C. J. Miller J. Brown H. Allan S. M. Pinteaux E. (2018). Changes in the secretome of tri-dimensional spheroid-cultured human mesenchymal stem cells *in vitro* by interleukin-1 priming. Stem Cell Res. Ther. 9 (1), 11. 10.1186/s13287-017-0753-5 29343288 PMC5773162

[B46] Song Y. Dou H. Li X. Zhao X. Li Y. Liu D. (2017). Exosomal miR-146a contributes to the enhanced therapeutic efficacy of interleukin-1β-primed mesenchymal stem cells against sepsis. Stem Cells 35 (5), 1208–1221. 10.1002/stem.2564 28090688

[B47] Tan C. Y. Lai R. C. Wong W. Dan Y. Y. Lim S. K. Ho H. K. (2014). Mesenchymal stem cell-derived exosomes promote hepatic regeneration in drug-induced liver injury models. Stem Cell Res. Ther. 5 (3), 76. 10.1186/scrt465 24915963 PMC4229780

[B48] Vandesompele J. De Preter K. Pattyn F. Poppe B. Van Roy N. De Paepe A. (2002). Accurate normalization of real-time quantitative RT-PCR data by geometric averaging of multiple internal control genes. Genome Biol. 3 (7), RESEARCH0034. 10.1186/gb-2002-3-7-research0034 12184808 PMC126239

[B49] Wedzinska A. Figiel-Dabrowska A. Kozlowska H. Sarnowska A. (2021). The effect of proinflammatory cytokines on the proliferation, migration and secretory activity of mesenchymal stem/stromal cells (WJ-MSCs) under 5% O2 and 21% O2 culture conditions. J. Clin. Med. 10 (9), 1813. 10.3390/jcm10091813 33919308 PMC8122617

[B50] Wolf M. Poupardin R. W. Ebner-Peking P. Andrade A. C. Blochl C. Obermayer A. (2022). A functional corona around extracellular vesicles enhances angiogenesis, skin regeneration and immunomodulation. J. Extracell. Vesicles 11 (4), e12207. 10.1002/jev2.12207 35398993 PMC8994701

[B51] Wu H. Fan H. Shou Z. Xu M. Chen Q. Ai C. (2019). Extracellular vesicles containing miR-146a attenuate experimental colitis by targeting TRAF6 and IRAK1. Int. Immunopharmacol. 68, 204–212. 10.1016/j.intimp.2018.12.043 30654310

[B52] Xu X. Gu S. Huang X. Ren J. Gu Y. Wei C. (2020). The role of macrophages in the formation of hypertrophic scars and keloids. Burns Trauma 8, tkaa006. tkaa006. 10.1093/burnst/tkaa006 32341919 PMC7175772

[B53] Yamada Y. Nakamura-Yamada S. Umemura-Kubota E. Baba S. (2019). Diagnostic cytokines and comparative analysis secreted from exfoliated deciduous teeth, dental pulp, and bone marrow derived mesenchymal stem cells for functional cell-based therapy. Int. J. Mol. Sci. 20 (23), 5900. 10.3390/ijms20235900 31771293 PMC6928984

[B54] Yao M. Cui B. Zhang W. Ma W. Zhao G. Xing L. (2021). Exosomal miR-21 secreted by IL-1β-primed-mesenchymal stem cells induces macrophage M2 polarization and ameliorates sepsis. Life Sci. 264, 118658. 10.1016/j.lfs.2020.118658 33115604

